# Novel approach for efficient resonance tracking in photoacoustic gas sensor systems based on a light-induced wall signal

**DOI:** 10.1016/j.pacs.2023.100495

**Published:** 2023-04-14

**Authors:** C. Weber, J. Kapp, J. Wöllenstein, K. Schmitt

**Affiliations:** a Department of Microsystems Engineering–IMTEK, Laboratory for Gas Sensors, University of Freiburg, Georges-Koehler-Allee 102, 79110, Freiburg, Germany; b Fraunhofer Institute for Physical Measurement Techniques IPM, Koehler-Allee 301, 79110, Freiburg, Germany

**Keywords:** Resonance tracking, Wall signal Resonant cell, Photoacoustic spectroscopy, Gas sensing

## Abstract

Photoacoustic gas sensing is a method suited for the detection of radiation absorbing molecular species in the gas phase. Due to the backgroand-free detection, it has considerable benefits in the measurement of very low concentrations down to the parts-per-trillion range. Yet in resonant systems, the resonance frequency depends on several parameters like temperature or gas composition and therefore must be continuously determined. In the present work, we propose a new method of tracking the resonance frequency using a photoacoustic signal generated at the walls of the resonant cell. The method has been evaluated with two different photoacoustic setups intended for the detection of NO_2_. We further propose an algorithm for finding the resonance frequency and evaluated the performance thereof. With this method, it is possible to detect the resonance frequency of a cylindrical and a dumbbell-shaped cell in less than two seconds and with an accuracy < 0.06% and < 0.2%, respectively.

## 1 Introduction

Photoacoustic (PA) gas sensing is the quantitative determination of radiation absorbing gas species based on the photoacoustic effect. The PA effect describes the conversion of the absorbed radiation energy into pressure. In case of temporally modulated radiation, the resulting fluctuating pressure can be detected as a soand wave [1]. As the amount of absorbed energy is proportional to the number of absorbing molecules and also to the soand pressure, PA gas sensing shows a linear correlation between gas concentration and soand pressure over several orders of magnitude [2,3]. Contrary to other absorption-based techniques, PA gas sensing is an offset free technique, meaning that in absence of the target gas, no signal is generated [4]. This offers a substantial advantage for detecting low concentrations, as, in an ideal case, the zero point is not disturbed by drift effects [5]. The signal strength of PA gas detectors depends on the radiation strength, the absorption coefficient, and the number of molecules of the species to be detected [6,7]. The acoustic signal becomes inherently weak for low concentrations down to the ppb (parts per billion) or ppt (parts per trillion) range. Thus, it vanishes easily in the noise of the soand transducer or the environmental backgroand. The transducer is typically a microphone, a cantilever, or a tuning fork [8]. Boosting the acoustic signal by resonant amplification increases the amplitude, which consequently lowers the detection limit [9]. With resonant amplification, a soand wave is generated in a gas cell where it is reflected in a constructively interfering manner, forming a standing wave. In the antinodes, the acoustic pressure is increased, and the signal-to-noise-ratio (SNR) is significantly improved.

This method is simple and therefore widely implemented. However, it has the significant disadvantage of making the measurement dependent on the resonance conditions. Especially in resonators, which have a high quality factor, the signal drops extremely even if the modulation frequency of the light source slightly deviates from the eigenfrequency of the PA cell [10]. This is further complicated by the dependence of the eigenfrequency on the speed of soand which in turn depends on temperature, pressure and on the molecular gas composition [11,12,13]. Physical parameters like temperature can be measured and used to compensate for the change in frequency [14]. However, in most applications the exact carrier gas composition (gas matrix) is unknown and/or varying. Therefore, changes of the eigenfrequencies cannot be theoretically estimated and it is thus necessary to determine them continuously in situ [15]. One simple approach is to deduce the current eigenfrequency from the photoacoustic measurement data itself. A concept reported in literature, that works in this manner, suggests the consecutive monitoring of the phase between excitation and the microphone signal. If a phase deviation occurs, the modulation frequency is adapted accordingly [16,17]. Although this concept works well for short and consecutive measurements, it has some drawbacks if the eigenfrequency changes significantly without intermediate measurements. In duty-cycled applications or for an initial measurement, other concepts might be better suited. Finding the resonance by a sweep of the modulation frequency is one example [18]. In this case, the modulation frequency of the radiation source is tuned over the practicable range while seeking for the signal maximum. The sweep can be performed prior to the actual measurement, but it can also be used for estimating the gas concentration [19]. Likewise, a chirp signal containing all the relevant frequencies can be used. With a Fourier transformation of.

the resulting acoustic signal, the resonance peak position can be calculated [20].

All principles mentioned above have the essential drawback that they rely on the photoacoustic signal generated in the target gas. For low concentrations close to the detection limit, the SNR of the signal is intrinsically poor. Thus, finding the resonance frequency based on this data becomes challenging. An inappropriate resonance frequency determination might add an additional measurement error and can in turn impair the detection limit.

Integrating an electromechanical soand transducer into the photoacoustic cell is a possible solution and is already an established method [21,22,23]. The transducer, most commonly a piezoelectric or electrodynamic loudspeaker, can deliver a very strong acoustic signal allowing to find the eigenfrequencies of the cell. Analogous to the determination of the measurement signal, this acoustic signal can be generated by means of a frequency sweep, a chirp procedure, or the evaluation of the pulse response of the cell.

Although all these principles provide highly reliable methods for finding the eigenfrequencies, they add some complexity to the resonant cell. Furthermore, the transducer might have a negative impact on the cell properties because its structure can pose a possible discontinuity in the acoustic impedance of the resonator.

To overcome these drawbacks, we propose a new method of finding the resonance frequency based on irradiating light onto the cell walls and using the resulting photoacoustic soand wave as basis for finding the eigenfrequencies. The method requires only a few additional components and can easily be integrated in most photoacoustic gas sensing setups.

## 2 Method

As with the photoacoustic effect in gasses, a photoacoustic wave is also generated when electromagnetic radiation with temporally varying intensity hits absorbing solids [24]. In photoacoustic gas measurement setups, this is usually an unwanted effect, as they rely on the generation of soand solely by the gas of interest. Additional wall or window-borne soand distorts the gas measurements and is therefore often referred to as *wall* or *window noise* [25]. To minimize such backgroand effects, a large variety of methods ranging from bafflers [26] over special cell geometries [27] to spectral light source modulation or differential resonator concepts [28] are described in literature.

We propose utilizing this normally unwanted effect by switching between two phases that differ largely in the amount of their wall- generated signal. In the photoacoustic gas measurement phase, the radiation is guided through the cell, preferably with no wall interaction. In a second phase, the tracking phase, the radiation is guided towards the wall, where it generates a strong acoustic wave independent of gas absorption. This signal can be used to accurately find and track the acoustic eigenmodes of the cell. Due to its photoacoustic generation, the signal should exhibit a linear frequency response without structural resonances present in electromechanical transducers.

If the same light source is used for both phases, the switching can be performed by moveable optics such as lenses or mirrors. Yet, a much simpler way is to add an additional light source that is solely used to generate the wall signal. Therefore, in this work the light from the source used for resonance tracking is referred to as *tracking radiation*, while the light used for the photoacoustic measurement is denoted as *measurement radiation*.

Light emitting diodes (LEDs) are ideal for generating the tracking radiation, as they are cheap, obtainable in small packages and easy to modulate by controlling their driving current. Yet also other sources like lasers or mechanically chopped continuous sources could be used instead.

With all the measures to avoid a soand generation on the walls in the measurement phase, the tracking radiation needs to be absorbed in such a way that these measures show a low or no effect on it. Otherwise, the signal strength and thus the SNR is drastically reduced. With low SNR, the resonance tracking is more prone to errors, or it might even be impossible. When bafflers or special cell geometries are used, it is usually sufficient that the tracking radiation hits the wall in a location where the measures are less effective. In the simplest case, the tracking light can be brought into the cell in a large divergent angle hitting a large part of the inner the surface sections. This usually includes parts where a soand generation is not effectively suppressed. Although this does not provide the largest possible signal, the constructive requirements are low, and no additional optics is needed.

Obviously, the radiation can also be focused on a part of the resonator surface, where the wall-noise counter measures are evaded particularly well. Ideally, the already existing optical elements for guiding the measurement radiation can be used for this purpose. Otherwise, the required components would increase the overall complexity.

In order to obtain a signal capable of stimulating the acoustic eigenmode of interest, the overlap integral between the soand source and the resonant mode is important [29]. Given that the soand emerges from the direct vicinity of the illuminated surface, the tracking radiation must also be absorbed in a place where the overlap integral with the target resonant mode is high.

The analysis of the obtained photoacoustic signal can be done similarly to the approaches that apply in systems with speakers. This includes methods like phase locking [23], broadband chirp excitation with spectral examination [30] or a frequency sweep in the time domain with amplitude analysis [31].

## 3 Experimental setups

**Fig. 1:**
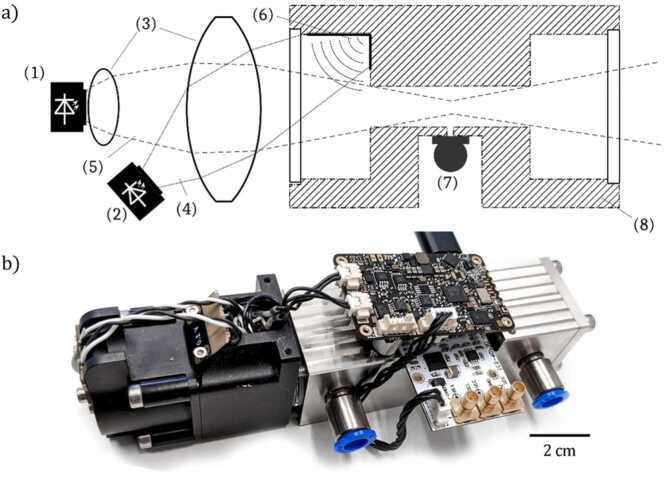
Schematic cross section of the tracking method employed in a dumbbell-shaped cell with (1) the measurement radiation source, (2) tracking radiation source, (3) optics, (4) tracking radiation, (5) measurement radiation, (6) absorptive coating and tracking signal generation, (7) microphone and (8) photoacoustic cell. The photo b) shows the setup that was used to investigate the tracking technique. The measurement radiation is focused through the resonator with two convex lenses, while the tracking radiation incides oblique to the beam axis and is focused onto the wall of the first buffer volume. As the buffer structure cannot eliminate soand that is generated in this area, the resonance can be excited from outside the resonator.

The performance of the proposed tracking principle was investigated with two different resonant photoacoustic cells, both suited to be used with divergent measurement radiation sources. The first one is a dumbbell-shaped cell, often referred to as H-cell, that is used in a variety of photoacoustic systems with LED [32] or laser [33] sources. Optimized for the large beam diameter of LEDs, the resonator has a diameter of 6 mm. With a length of 40 mm, its first acoustic mode is located at about 4 kHz. The open ends of the resonators are created by buffer volumes, that are 20 mm in length and diameter with windows to the outside. This cell and its optical setup are outlined in Fig. 1.

The radiation for the photoacoustic measurement is focused through the resonator by two convex lenses with minimal incidence onto the inner surface of the resonator. A small amount of radiation might hit the end face of the buffers near the resonator entrance, but the soand generated there is effectively suppressed by the buffer layout. The center of the resonator would be the ideal place for the tracking radiation to hit the inner surface of the cell. However, the aspect ratio of the cell prevents divergent radiation from an LED from being effectively focused on this area. Alternatively, the tracking radiation is guided into the cell at an angle and hits part of the side surface of the first buffer and its end face. Soand that is generated there is only partially suppressed by the buffer and can therefore indirectly stimulate resonance. By placing the tracking LED diagonally to the optical axis, the second lens acts as focusing element for it, eliminating the need for additional optics.

**Fig. 2:**
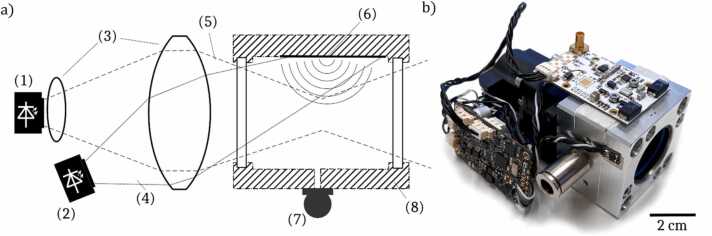
Schematic cross section of the tracking setup in a cylindrical PA-cell with (1) the measurement radiation source, (2) tracking radiation source, (3) optics, (4) tracking radiation, (5) measurement radiation, (6) absorptive coating and tracking signal generation, (7) microphone and (8) photoacoustic cell. b) shows a photograph of the cylindrical cell used in the investigations. Like in the dumbbell-shaped cell, the measurement radiation is focused through the cell while the tracking radiation passes one of the lenses oblique and is focused onto the cell’s wall. The radial resonance’s displacement node on the wall is shifted by 180° compared to the displacement node in the center of the cell, which leads to an additional phase shift when exciting the resonance with the tracking radiation.

The second resonant cell used for the experiments is intended for measurements using a radial acoustic mode. Its structure is similar to other resonant cells for radial modes, reported in literature [34], [35]. It exhibits a cylindrical shape with a length of 18 mm and a diameter of 20 mm. As shown in Fig. 2, this cell uses an optical arrangement of two lenses, analogous to that of the dumbbell cell. However, the wide diameter of the cylinder allows for a much larger spot size and a closer focus, allowing more light to pass through the cell. The tracking LED can also be arranged in an oblique angle in which the light is focused onto the inner lateral area.

Besides the cells geometry and the optical layout, the other components of the photoacoustic investigation systems are identical. Both systems were designed for the photoacoustic detection of nitrogen dioxide (NO_2_). In the wavelength range from 300 nm to 600 nm, NO_2_ shows a strong absorption with a peak at 390 nm [36]. A light source emitting at this central wavelength would result in the strongest NO_2_-signal. However, radiation with a wavelength below 415 nm can cause photodissociation [37]. Thus, LEDs with central wavelengths of 450 nm are often used for detecting NO_2_ in the visible range. The LEDs used in our systems are GD CSSRM2.1 by Osram OS, Netherlands, with an optical power of 2 W and a spectral bandwidth of 18 nm and a dominant wavelength of 450 nm.

The emission spectrum of the tracking LED should not overlap with the absorption bands of the target gas or other atmospheric components. Especially in the cylindrical cell, additional absorption in the gas phase would reduce the overlap integral. In the range from 850 nm to 875 nm, the atmosphere has a window of minimal absorption [38]. This includes NO_2_, that also does not absorb significantly in this range [39]. Therefore, a near infrared LED with a central wavelength of 860 nm (SFH 4718 A – Osram OS, Netherlands) was employed as source for the tracking radiation. It has an optical power of 660 mW at 1 A. Both LEDs were mounted on black anodized aluminum holders, which were also used for mounting the lenses. The acoustic cells were made of aluminum (EN AW6060), which is reflective in the relevant wavelength range. Therefore, the surface areas on which the tracking radiation impinges were coated with a thin layer of absorbing black color dye. The coating increases the absorption, enhancing the obtained tracking signal. Both designs used a bottom port MEMS microphone (ICS-40720, TDK, Japan), mounted on a printed circuit board, which also contains the analog signal processing. The analog signal was band pass filtered between 100 Hz and 20 kHz and amplified by a factor of 100. The digitalization and data computing were performed on a customized processing board. It incorporated a microcontroller (PSoc6 - Infineon Technologies AG, Germany), signal digitalization, waveform generation, LED drivers, power conditioning and a USB interface for the connecting to a host PC. In Fig. 1 and Fig. 2, both cells are shown with the attached optics block, the analog preamplifier board (white PCB) and the processing board (black PCB).

The microphone data was acquired at 1 MSps, and digitally filtered by a finite impulse response lock-in-filter with a variable time constant. As reference for the lock-in algorithm, the system uses the generated sinusoidal waveform, which is also the source for driving the LED current profile.

## 4 Experimental

### 4.1 Resonance profile matching

For the feasibility of the proposed tracking method, it is crucial that the frequency responses of the measurement and the tracking phase yield identical peak frequencies. As the concept relies on the photoacoustic excitation of the soand wave in different spatial positions, the overlap integral between the resonance profile and the soand source will differ. The resonance frequency will only be foand if the measurement radiation and the tracking radiation excite the resonance of interest efficiently and without any frequency deviation.

In a first investigation, the amplitude and phase response for measurement and tracking were recorded over a large frequency range for a qualitative comparison of the respective frequency responses. Successively, the measurement and tracking source were swept from 500 Hz to 25 kHz, while the filtered amplitude response was recorded. The interval was aroand 15 Hz, while the integration time constant of the filter was set to 250 ms, resulting in an overall sweep time of 400 s. In order to generate a significant measurement LED signal, both cells were purged at 0.1 l/min with synthetic air containing 20 ppm of NO_2_.

**Fig. 3:**
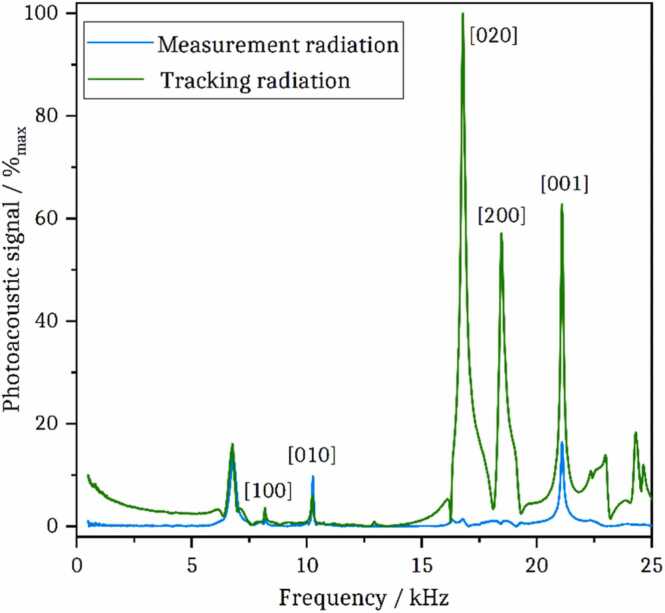
Frequency-dependent response of the cylindrical cell during excitation by absorption of the target gas with the measurement radiation (blue) and the excitation on the wall with the tracking radiation (green). The radial mode [001], which is the target mode, is effectively excited with both sources.

The result of the measurement for the cylindrical cell is shown in Fig. 3. In the tracking signal, the first two azimuthal modes [010][020], the first two longitudinal [100][200] and the first radial mode [001] are visible. The first azimuthal mode is not effectively excited by the tracking radiation, as it exhibits a pressure node at the microphone. The second azimuthal and the first radial modes have a large overlap integral with the area where the tracking radiation incides and therefore yield a pronounced resonance response. With the optics that were utilized, the tracking radiation is focused more on the far end of the cylinder seen from the entrance window. Thus, the second longitudinal mode also shows a strong signal amplification. Below 6 kHz, the tracking radiation generates a significant non-resonant signal that increases with lower frequency. It occurs only in the tracking case, and not with the measurement radiation, which indicates that it is not attributed to acoustics. It is most probably caused by direct radiation absorption of the microphone being sensitive to the strongly scattered light fraction in the tracking case. The tracking signal also reveals some additional modes that cannot easily be attributed to the cylindrical geometry. Presumably they belong to resonances influenced by the microphone connection duct or the gas connectors of the cell.

The absorption of the measurement radiation excites the photoacoustic wave with almost perfect axial symmetry. The resulting low overlap integral with the azimuthal and the longitudinal modes makes it hardly possible for them to be stimulated. The first azimuthal mode, however, is distinctive to a certain extent, likely due to some asymmetry. As expected, the radial mode exhibits a high resonant signal when measuring.

**Fig. 4:**
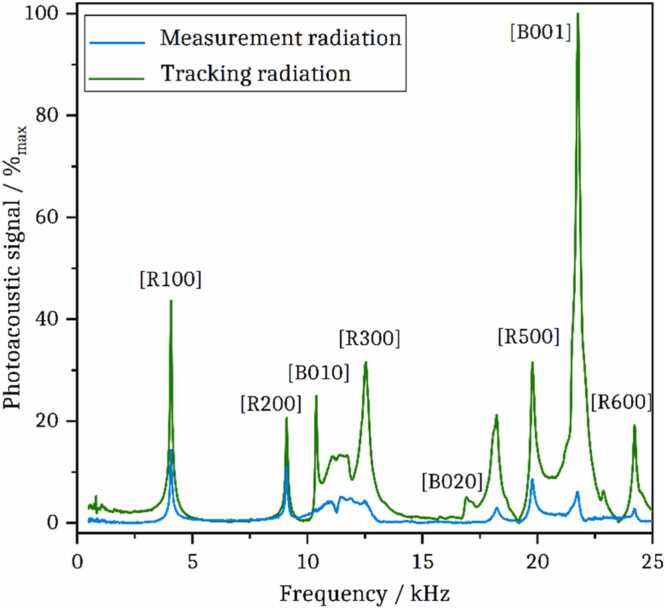
Amplitude response obtained from the dumbbell-shaped cell when excited with the measurement radiation (blue) and the tracking radiation (green). The relevant resonances are denoted with a B if they originate from the buffer and with an R if they belong to the resonator.

Compared to the cylindrical cell, the frequency response of the dumbbell-shaped cell shows more resonant peaks, as depicted in Fig. 4. The target mode is the lowest resonance that occurs in the tracking signal. Aside from the higher harmonic longitudinal modes in the resonator, there are also modes visible belonging to the buffers. The modes, an azimuthal buffer mode and a radial buffer mode, occur at frequencies similar to those of the cylindrical cell. As the dimensions of the buffer volumes roughly match the overall size of the cylindrical cell, this finding is in line with previous results. Some of the higher longitudinal resonator modes, visible in the tracking data, extend into the buffer volumes, so they deviate from their theoretical value. The curve of the measurement radiation shows that it can primarily excite the resonator modes. Like in the cylindrical cell, the azimuthal modes are hardly stimulated as a result of the axial symmetry. The radial mode in the buffer volumes is also weak, especially compared to the tracking signal. The lowest longitudinal resonator mode, being the target mode, exhibits the strongest measurement peak in the considered frequency range.

The results show that in both cases, the target mode was efficiently excited by the wall-absorbed tracking radiation, while the measurement radiation also yields a strong signal on the same resonant mode.

Beside the ability to be excited by measurement and tracking radiation, it is essential for a tracking algorithm that the resonance peak frequencies do not deviate between both phases. For investigating whether this condition was met, another sweep with a higher frequency resolution of 4 Hz was recorded with both systems. The range was reduced to 3.8–4.4 kHz for the dumbbell-shaped cell and 20.8–21.4 kHz for the cylindrical cell. The filter averaging time was increased to 500 ms, which increases the SNR.

**Fig. 5:**
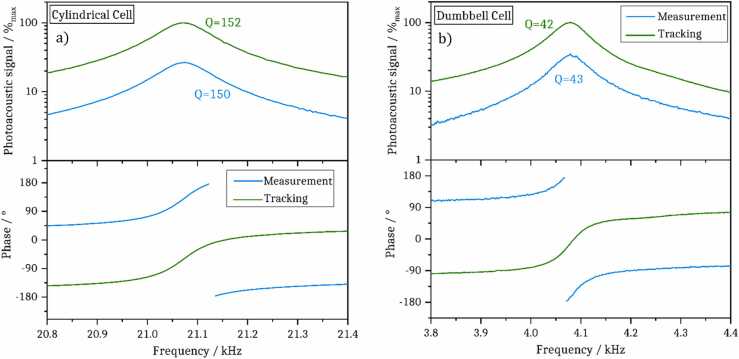
Detailed comparisons of the resonance profiles in amplitude and phase near the target resonance modes of the cylindrical(a) and the dumbbell-shaped cell (b). For a better analysis of the amplitude shape regardless of the signal strength, the amplitude is plotted logarithmically. For both cells, the resonance peaks of measurement and tracking deviate less than the used frequency intervals. The phase shift between tracking and measurement is almost 180° in the cylindrical cell and about 152° in the dumbbell cell. The quality factors Q in both the measurement and the tracking case are basically equal.

The results of both systems are shown in Fig. 5. In the cylindrical cell, the resonance shape of the tracking matches the one when excited with the measuring radiation almost perfectly. Only small shape deviations are visible on both ends of the sweep range. The result of the dumbbell-shaped cell is very similar. However, the tracking signal seems to be superimposed with a linear effect, raising the signal at the low end of the sweep. This is probably caused by direct radiation absorption by the microphone, that is most effective at low frequencies. In both systems, no frequency deviation was observed between the peaks of the measurement and the tracking curve. A possible mismatch is clearly smaller than the frequency intervals in the sweep of 4 Hz. It can therefore be assumed that the resonant frequencies foand with the tracking radiation are identical to those in the photoacoustic measurement. For both cells, the quality factors, are basically equivalent in the tracking and the measurement case. So the resonant losses do not differ between tracking and measurement.

As the tracking radiation induces the radial mode on the lateral surface of the cylinder, its overlap integral has an opposite sign compared to that of the gas excitation in the center. Therefore, the phase of tracking and measurement curves should exhibit a phase shift of 180° to each other. As depicted in Fig. 5, the phase difference of 183° agrees very well with the theory. In the signal of the dumbbell cell, the 152° phase shift is a rather arbitrary value. It can be explained by the tracking radiation being absorbed outside the resonance cylinder. The additional path to the resonator causes a delay and a subsequent phase shift.

### 4.2 Tracking algorithm

**Fig. 6:**
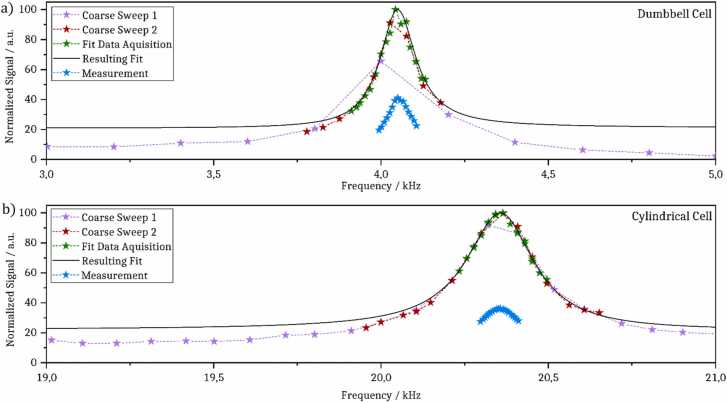
Graphical representation of the different steps performed by the tracking algorithm in the dumbbell-shaped setup (a) and the cylindrical setup (b). In a first iteration, a raw sweep (violet data points) was performed. By a maximum evaluation, the resonance position is determined, and a second sweep (red data points) was performed for a coarse peak finding. Afterwards, in a third iteration, the data points represented in green were acquired, and a resonator model was fitted to them (black line). The peak position was extracted from the fit. Datapoints in blue show a subsequently recorded confirmation sweep with the measurement radiation, pointing out the position of the true resonance maximum in the measurement. The data are from an NO_2_ measurement containing 20 ppm at normal conditions in synthetic air as carrier gas.

With the resonance peak in the tracking signal coinciding with the maximum in the photoacoustic gas measurement, a simple algorithm for resonance tracking can be used. Basically, a sweep of the tracking signal over the range where the peak is expected and a subsequent search for the maximum is sufficient. However, this method would be prone to noise artefacts. As a sweep consists of a variety of individual datapoints at distinct frequencies, it would also require small intervals to obtain a value close to the maximum of the resonance. Scanning a large range with small intervals and a high SNR is time consuming and thus not suited for rapid changes. For a faster determination of the resonance frequency, we propose a data acquisition in three iterative sweeps with decreasing intervals, followed by the fitting of a universal resonance function. The position of the peak is then derived from the fit parameters. In the first iteration, data points are obtained by sweeping the frequency of the tracking radiation in intervals of up to 200 Hz. This step is performed over the whole range where the peak might be located. Depending on the gas matrix and possible temperature range, the peak can shift by a large factor, requiring that the range of the first sweep must be selected accordingly. This procedure is depicted for both photoacoustic systems by the purple datapoints in the graphs in Fig. 6. In the first iteration, the SNR is of minor importance. However, the peak-to-peak noise needs to be lower than twice the minimum height of the peak to be foand. An interval equivalent to the full width half maximum (FWHM) of the peak is sufficient for not missing it. By simply searching the maximum amplitude of this sweep, the approximate location of the peak can be determined. If higher resonance peaks are present in the frequency range of interest, the parameters of the sweep have to be adjusted so that all major peaks are detected, and the peak of interest can be differentiated by its position relative to the others.

After the approximate resonance frequency is foand, a second sweep is performed using a smaller interval between the data points to obtain a better approximation of the peak position. The aim of this step, shown in red in Fig. 6, is to find a starting point for a symmetric data acquisition in the next iteration. The interval has about 10–30% of the width of the peak at half its height. This is adequate for a rough estimation.

In the third iteration, datapoints are acquired symmetrically aroand the approximate peak position. The points should have a small interval, and the range should preferably cover the upper two thirds of the peak. The other data points do not carry much information on the position of the peak and are therefore not desirable. By fitting a universal resonator model to the data, the shape of the peak is determined. The model that was used is based on the Lorentzian function, which is often used to describe resonance phenomena [40,41]. It has been modified so that the width of the curve has a first order polynomial dependency on the frequency. This corresponds to a linearly changing dampening and is necessary, as otherwise a likely peak asymmetry would lead to large position errors. The function has the form as shown in Eq. (1).

(1) $$y(f)=V+A\cdot \frac{{{(\gamma }_{L}\left(f-f_{0}\right){+\gamma }_{0})}^{2}}{{{(\gamma }_{L}\left(f-f_{0}\right){+\gamma }_{0})}^{2}+{\left(f-f_{0}\right)}^{2}}$$

*V* represents the offset, *A* the amplitude, *f*_*0*_ the peak position and γ_L_ and γ_0_ corresponding to the linear and constant part of the width parameter.

The model is dimensioned in such a way that the peak position and the flanks of the resonance curve are described particularly well. However, it does not represent the curve features distant from the peak with comparable precision. As shown in Fig. 6, this leads to a large deviation between data and fit in the remote parts, yet this is not significant for locating the peak position. The model is also favorable in terms of fast data processing, as the function itself as well as its derivatives consist of simple mathematical operations. This allows the Levenberg-Marquardt algorithm to be executed efficiently on the microprocessor itself.

After the resonance frequency is determined, the actual photoacoustic measurement can be performed at the correct modulation frequency.

### 4.3 Tracking characterization

Further investigations were carried out with both systems to verify the performance of the proposed tracking mechanism. During the measurement, the resonance frequencies were artificially changed by adding different concentrations of CO_2_ to the gas mixture. In pure CO_2_ the soand velocity at 280 m/s [42] is approximately 15% lower than in air (330 m/s) [43]. Subsequently, the concentration of CO_2_ alters the speed of soand in the gas mixture depending on the amount and the resonance profiles shift accordingly. By varying the CO_2_ content between 0% and 40%, an overall frequency shift of about 8% was achieved.

The gas concentrations were established by a gas mixing station at a flow of 200 ml/min. The CO_2_ concentration was changed in steps of 5%. The carrier gas was synthetic air consisting of 80% nitrogen and 20% oxygen. To generate a strong measurement signal, 20 ppm NO_2_ were added to the mixture. Its concentration was kept constant during the measurement, independent of the CO_2_ concentration. These Measurements were performed ander lab conditions at 22(2) °C and at atmospheric pressure of 1050(70) hPa.

Both sensor systems were connected in series in the gas flow, assuring comparable flow conditions and concentrations. With inner volumes of approximately 7 ml (dumbbell shaped cell) and 15 ml (cylindrical cell), the cells are small enough to establish a concentration equilibrium in less than one minute.

The tracked resonance frequency was obtained by the tracking procedure shown in Fig. 6, with all processing done in the sensor hardware. For the cylindrical cell, the lock-in filter time constant was set to 10 ms for all data points obtained with the tracking radiation. The coarse sweep consisted of 39 data points ranging from 18.5 kHz to 22.3 kHz. The following fine sweep was formed by 15 points while the data for the fit contained 20 points. Including the computation time for the waveform output, the complete data acquisition took about 1.1 s. Depending on the data quality, the curve fit added another 60–200 ms, resulting in an overall resonance tracking time of well below two seconds. For the examination of the deviation between the tracked frequency and the *true* resonance frequency, a 20-point measurement radiation sweep in a range of ± 50 Hz aroand the tracked frequency was performed after the tracking. The resonance frequency from the measurement is also referred to as *true*, as it is the target frequency for the measurement. For obtaining high quality data, the time constant of the lock-in filter was set to 1000 ms in this sweep. The entire process was repeated continuously in the gas measurement. For each repetition the tracking is started from scratch, independent of the previous data set. The tracked resonance frequency as well as the peak position in the NO_2_ measurement were then transferred to a PC for further evaluation.

The characterization of the tracking in the dumbbell cell was performed in an almost identical manner, but self-evidently utilizing other frequency ranges and intervals for the acquired tracking data. The coarse sweep consisted of 15 points ranging from 2 kHz to 5 kHz, while the subsequent sweep had 10 datapoints and the fitting data 15. Due to the much lower resonance frequency of the dumbbell cell, the filter requires a longer time constant for the same off-band noise suppression. It was therefore set to 20 ms. Along with the lower number of points, this resulted in a data acquisition time of 1150 ms, which is comparable to that of the radial system. The fit computation also added up to 200 ms, which kept the overall tracking time below two seconds as well. The sweep with the measurement radiation is performed analogous to the procedure in the cylindrical cell measurement.

**Fig. 7:**
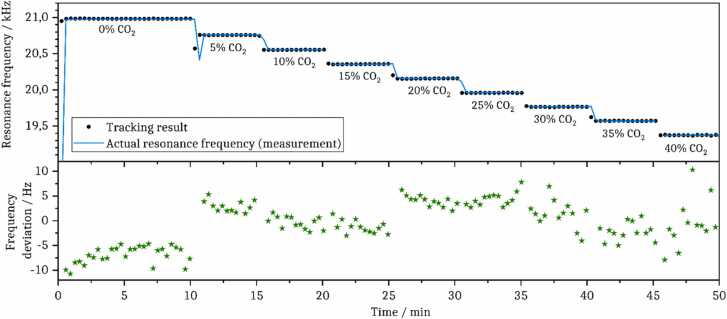
Result of the tracking characterization of the cylindrical cell. The speed of soand of the carrier gas (synthetic air), and thus the resonant frequency of the cell, was changed stepwise by adding different amounts of CO_2_. In the upper panel, the blue line represents the measured true resonance frequency obtained from a sweep with the measurement radiation. The black dots show the resonance frequency determined from the wall-generated signal of the tracking radiation. The lower panel depicts the deviation between both frequencies in one measurement iteration. When the CO_2_ concentration changes rapidly, the determination of the true resonance frequency occasionally fails, which is represented by a missing blue line.

The result of the tracking characterization for the cylindrical cell is shown in Fig. 7, in which the blue line depicts the true resonance frequency that is extracted from the high SNR sweep with the measurement LED. As expected, it drops approximately by 250 Hz per CO_2_ step due to the decrease of the speed of soand in the gas mixture. The black dots represent the resonance frequency that was determined by the proposed tracking algorithm with the wall signal generated by the tracking radiation. During the entire measurement, the tracking algorithm could follow the fast changes of the resonance frequency. With its fast data acquisition, it is even capable of finding the resonance in the moment of a strong change in speed of soand. The sweep performed with measurement radiation takes about 25 s, which is slower than the change of the CO_2_ concentration. Therefore, the determination of the true resonance frequency at the times of fast changes is not always possible or prone to errors. Yet the tracking itself always succeeded.

The frequency deviation between the tracked and the true resonance peak position is shown in the lower panel of Fig. 7. A systematic deviation of up to 7 Hz, that seems to be correlated with the CO_2_ concentration, is noticeable. This is most likely due to the discretization of possible LED modulation frequencies. For computation efficiency, their interval is limited to 0,05% of the base frequency when acquiring the tracking samples. At aroand 20 kHz this leads to a discretization of 11 Hz steps (this is also visible in the lower panel of Fig. 6, in which the green datapoints are partially sampled at the same frequencies, because of this rough spacing). Accordingly, by decreasing the possible output frequency spacing, this effect can be circumvented at the cost of some computation time. The systematic error accounts on average for about 6 Hz or 0.03% of the deviation. Thus, this error is low enough to be acceptable for most applications.

Another visible effect is that the tracking quality seems to depend on the CO_2_ concentration. The noise induced dispersion rises from ± 3 Hz at low CO_2_ concentrations to ± 6 Hz at 30%. This can be explained by the CO_2_ dampening the resonance, thereby reducing the signal strength, which consequently lowers the SNR. The standard deviation increases from 1.2 Hz to 2.5 Hz. However, this has only a small effect on the overall tracking error and the resonance frequency is reliably foand.

Even without a modification of the frequency output spacing, the proposed tracking method can find the resonance peak with a maximum overall error of ± 12 Hz or ± 0.06% in the cylindrical cell system. Given this frequency uncertainty, the subsequent error of the gas measurement signal amplitude would be at maximum 0.5%. Usually, the relative precision of photoacoustic measurement systems is limited to several percent due to the unknown thermal properties or the relaxation behavior of the gas mixture [44,45]. Thus, the additional tracking error is negligible in most cases.

**Fig. 8:**
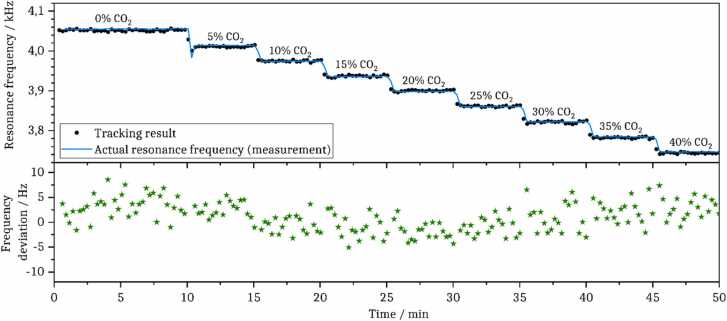
Result of the tracking investigations on the dumbbell-shaped cell. The speed of soand of the carrier gas (synthetic air), and thus the resonant frequency of the cell, was changed stepwise by adding different CO_2_ concentrations. The photoacoustic signal excited by the measuring radiation is based on NO_2_ absorption (20 ppm). Like in the graph for the cylindrical cell, the blue line represents the true resonance frequency while the black dots illustrate the tracking result obtained from the wall generated photoacoustic signal. The lower panel shows the deviation between the frequencies in one iteration.

In Fig. 8, the measurement data of the equivalent investigations on the dumbbell cell system is depicted. With the lower resonance frequency and the comparable time budget for the tracking, the obtained data shows a higher relative dispersion on the frequency deviation. Still, the tracking can detect the true resonance frequency without larger errors. A systematic offset between tracking and measurement is suspected, but it is much lower than the noise-induced deviation and therefore not clearly quantifiable. The standard deviation of the frequency was about 2.5 Hz in the whole measurement and the highest overall deviation was 8.5 Hz. Compared to the cylindrical cell, the resulting relative deviation of 0,2% is more substantial, yet its impact is mitigated due to the lower quality factor of the dumbbell cell. The maximum gas measurement error introduced by a mismatch of 8.5 Hz would be in the range of 1.5% of the measurement signal. This is sufficient for most applications, however, if more precision is needed, the time constants of the tracking or the intensity of the emitted radiation of tracking could be increased.

## 5 Conclusion

A method for determining the resonant frequency of photoacoustic gas sensing cells is proposed in which the wall of the cell is illuminated with a second light source, in this case an LED. The light absorbed by the wall of the photoacoustic resonator generates an acoustic wave, which makes it possible to obtain information about the acoustic properties of the cell without absorption of the target gas. In the proposed method, it is important that the excitation of the wall absorption and the measurement yield the same acoustic resonance profiles, especially for the respective target modes. Two demonstrators for this kind of tracking mechanism were developed, one cylindrical photoacoustic cell, and a dumbbell shaped one. For both, the implementation of the additional LED required minimal effort, as the optics assigned for the measurement could also be used for the tracking radiation. Compared to setups with soand transducers placed in the photoacoustic cell, our proposed method did not require modifying the cell itself.

The experimental characterization showed an excellent match of the resonance profiles of the measurement and resonance tracking for both setups. Particularly on the target modes, no measurable deviation between the resonance peak maxima of both methods was observed. By using a sweep algorithm with multiple iterations and a subsequent fitting of a modified Lorentzian function, a method for analyzing the obtained data was proposed. It enabled finding the resonance frequency in less than 1.5 s for both sensor systems. In experiments in which the resonance frequency was modified intentionally by the gas mixture, the proposed method was able to track the resonance without larger errors. In the system with the cylindrical cell, the determined frequency deviated at maximum by 12 Hz from its measured value, which corresponds to an error of below 0.06%. The induced error from the frequency mismatch on the gas concentration can be estimated to stay below 0.5%.

The results of the dumbbell-shaped cell showed a maximum deviation of 8.5 Hz, or 0.2% between the tracked frequency and its measured value. Despite this deviation, the resonance frequency was foand in a reliable manner, and the additional concentration error of gas measurements still could be estimated to stay below 1.5%. The results indicate that the presented tracking method is applicable in various resonant photoacoustic gas detection applications, replacing more complex solutions like dedicated speakers or less powerful methods such as pure measurement signal evaluation.

## Declaration of Competing Interest

The authors declare that they have no known competing financial interests or personal relationships that could have appeared to influence the work reported in this paper.

## Data Availability

The data that has been used is confidential.
